# Relationship between triglyceride glucose-body mass index baselines and variation with future cardiovascular diseases risk in the middle-aged and elderly individuals

**DOI:** 10.3389/fendo.2025.1514660

**Published:** 2025-01-27

**Authors:** Junpeng Qiao, Xueyu Chen, Jinhong Pang, Haicheng Fei, Zhang Liu, Fang Cheng, Qiaoqiao Chen, Yingying Zhao, Fengxue Shi, Hongying Jia, Weiwei Chi

**Affiliations:** ^1^ Department of Epidemiology and Health Statistics, School of Public Health, Cheeloo College of Medicine, Shandong University, Jinan, China; ^2^ Department of Clinical Skills Center, School of Clinical Medicine, Shandong First Medical University & Shandong Academy of Medical Sciences, Jinan, Shandong, China; ^3^ The School Hospital, Shandong University, Jinan, Shandong, China; ^4^ National Administration of Health Data, Jinan, Shandong, China

**Keywords:** triglyceride glucose-body mass index, cardiovascular disease, K-means clustering, CHARLS, middle and elderly age

## Abstract

**Background:**

Cardiovascular diseases (CVDs) are gradually becoming the leading cause of morbidity and mortality among chronic non-communicable diseases. Previous studies have found that the TyG index is an effective alternative indicator for insulin resistance (IR) and is associated with cardiovascular events. Additionally, obesity directly or indirectly increases the risk of developing CVDs. Up to now, studies on the combined effects of these factors are insufficient, and the conclusions are not yet consistent. This study aims to analyze whether the baseline levels and fluctuations of triglyceride glucose-body mass index (TyG-BMI) are associated with the incidence of CVDs and their subtypes in a prospective cohort of middle-aged and elderly individuals.

**Methods:**

The data for this study were obtained from the China Health and Retirement Longitudinal Study (CHARLS), which is an ongoing nationally representative prospective cohort study. After excluding participants with partially missing variables that could affect the study results, this study ultimately included 7,072 participants, with data records spanning from 2011 to 2020. The exposures were TyG-BMI and the change in TyG-BMI from 2011 to 2015. The TyG-BMI index was calculated as TyG index multiply BMI. The change of TyG-BMI was categorized using K-means clustering and baseline TyG-BMI was grouped based on quartiles. We used Cox proportional hazards models to evaluate the relationship between baseline quartiles of the TyG-BMI index and its variability with CVDs and their subtypes.

**Results:**

Among the 7,072 participants (mean age of 59.1 ± 9.3 years), 3330 (47%) were male. During an average follow-up of 7.1 years, 1,774 (25.1%) participants developed new-onset cardiovascular diseases. After stratification by baseline TyG-BMI quartiles, higher TyG-BMI levels were associated with an increased risk of CVDs, The hazard ratio (HR) and 95% confidence interval (95% CI) for the highest quartile group were 1.69 (1.44-2.00). After adjusting for potential confounding factors, compared to participants with consistently low TyG-BMI levels, those with moderate TyG-BMI levels and a slowly increasing trend had an HR of 1.27 (95% CI 1.10-1.47), while those with the highest TyG-BMI levels and a slowly decreasing trend had an HR of 1.52 (95% CI 1.26-1.83).

**Conclusion:**

Material changes in the TyG-BMI are independently associated with the risk of CVDs in middle-aged and elderly individuals. Detecting long-term changes in the TyG-BMI may aid in the early identification of high-risk individuals and help prevent the occurrence of various cardiovascular diseases.

## Background

CVDs are gradually becoming the most common cause of morbidity and mortality among chronic non-communicable diseases in both China and globally (1–5). According to reports, in 2019, the number of deaths from cardiovascular diseases worldwide reached 19 million (6). Although some studies indicate that the occurrence of CVDs is associated with various risk factors such as age, obesity, hypertension, diabetes, severe stress experiences, and rheumatoid arthritis, etc (7–9), recent research has found that individuals without these risk factors may still be at risk for CVDs (10). Therefore, there is still a lack of scientifically reliable, direct, and simple indicators to effectively and sensitively alert individuals to the severe risk of CVDs (11).

Insulin resistance (IR) refers to a pathophysiological condition in which cells fail to respond normally to insulin. IR as a key factor in metabolic disorders, triggers the onset of CVDs (12, 13). It not only amplifies the correlation between traditional factors such as obesity, dyslipidemia, hypertension and CVDs, but also increases the risk of CVDs by influencing non-traditional factors, such as microalbuminuria (14, 15). The hyperinsulinemic-euglycemic clamp (HEC) is considered the gold standard for assessing IR (16); however, this method is complex and expensive, limiting its widespread clinical application. As an economically effective alternative for assessing IR, the triglyceride-glucose (TyG) index can be easily obtained from routine clinical laboratory tests, and research has demonstrated its association with the incidence and progression of CVDs (17, 18).

Recently, a study found that combining the TyG index with BMI as a reliable alternative indicator for the early detection of insulin resistance is effective. This measure is calculated by multiplying the TyG index by BMI, incorporating multiple clinical indicators such as BMI, blood glucose, and lipid levels, thereby enhances its predictive ability (19). Moreover, studies have shown that TyG-BMI has been significantly associated with diabetes, kidney disease, and hypertension (20, 21). Although early studies have provided valuable insights, there is still controversy regarding the association between TyG-BMI and CVDs. Therefore, this study aims to investigate the relationship between baseline TyG-BMI and its variability with the incidence of CVDs in middle-aged and elderly populations.

## Materials and methods

### Study design and population

The CHARLS project aims to collect a set of high‐quality micro-data representing households and individuals aged 45 and above in China, so as to analyze the aging of Chinese population and promote interdisciplinary research on aging. The CHARLS is an ongoing survey with assessments conducted every 2 to 3 years, and it has released five waves data of national to date. The survey includes: basic demographic information of the respondents and their family structure, health status of the respondents, medical service utilization and insurance, employment, income, consumption, assets, retirement and pension, and so on. Additionally, CHARLS includes physical measurements and blood sample collection of the respondents. The CHARLS data can be freely downloaded from the official website (https://charls.pku.edu.cn/index.htm).

**Figure 1:**
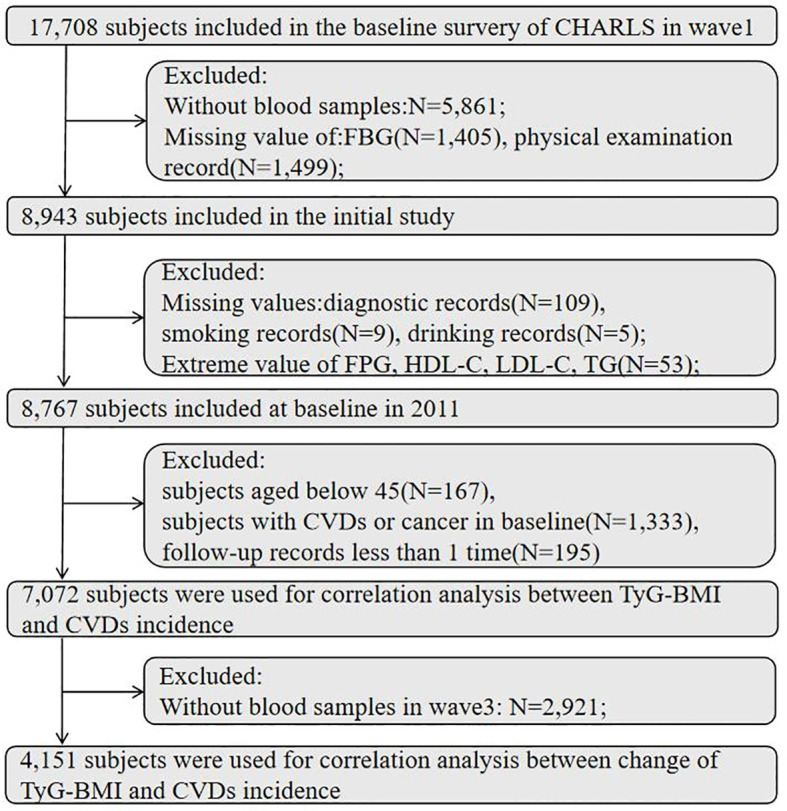
Flowchart of the study participants selection. FBG, fasting blood glucose; TG, triglyceride; HDL-C, high-density lipoprotein cholesterol; LDL-C, low-density lipoprotein cholesterol; CVDs, cardiovascular diseases.

Our research is a prospective cohort study based on the CHARLS database. This study utilized data from five waves. At baseline (wave1), a total number of 17,708 respondents completed a face-to-face computer-assisted personal interview, and the exclusion criteria were as follows: without blood samples; missing value of fasting blood glucose (FPG), triglyceride, body mass index; diagnosed with cardiovascular disease; with cancer; and having fewer than one follow-up record. A number of 7,072 participants were remained at baseline after exclusion criteria, and 4,151 subjects were used for correlation analysis between the variability of TyG-BMI and CVDs incidence. The detailed flowchart is shown in **Figure 1**.

### Diagnosis of CVDs

The outcome was the incidence of CVDs, including stroke and chronic heart disease (CHD). The diagnostic criteria for these diseases were: self-reported physician-diagnosed condition and currently use of medication for treating the diseases.

### Assessment of TyG-BMI

The TyG-BMI index was calculated as follows: TyG index = ln [TG (mg/dL) × FBG (mg/dL)/2]; BMI = weight (kg)/height (m²); TyG-BMI index = TyG index × BMI. The Baseline TyG-BMI index was calculated by TyG index and BMI in baseline (2011).

### Covariant definition

The data of this study were collected by investigators recruited and trained by the CHARLS research team. Demographic backgrounds, health status, anthropometric information, and blood data were identified and matched by a unique ID code.

The participants health record information included age, gender, education level, marital status and personal living habits. Education level was divided into “primary school and below”, “middle school” and “high school and above”. According to the questionnaire, marital status was divided into married and unmarried. Smoking and drinking habits were classified as “current smoking, former smoking, never smoking”, “current drinking, former drinking and never drinking”.

Participants meeting any one of the following criteria were considered to have diabetes: FBG≥7.0mmol/L(126mg/dL), self-reported doctor-diagnosed diabetes, or currently use of anti-diabetic medication.

Subjects who meet one of the following criteria were considered to have dyslipidemia: total cholesterol (TC) ≥ 6.2mmol/L, high-density lipoprotein cholesterol (HDL-C) < 1.0 mmol/L, low-density lipoprotein cholesterol (LDL-C) ≥ 4.1 mmol/L, or triglyceride (TG) ≥ 2.3 mmol/L. Additionally, self-reported doctor-diagnosed dyslipidemia or currently using lipid-lowering medication were also considered to have dyslipidemia.

Used an Omron HEM-7200 sphygmomanometer to measure the blood pressure in sitting position, and use standard measurement method to examine the blood pressure three times at an interval of 45 seconds, and take the average blood pressure value of the three measurement results as the final blood pressure. Hypertension was defined as systolic blood pressure (SBP)≥140mmHg, diastolic blood pressure (DBP) ≥ 90mmHg. We also classify participants who were diagnosed as hypertension by doctors or currently used antihypertensive drugs as hypertension group.

### Statistical analysis

Data analysis used various statistical methods. Data was tested for normality using the Kolmogorov–Smirnov test method. Mean and standard deviations (mean ± SD) were used to describe continuous characteristics, and percentages and frequencies (N, %) were used to describe the categorical characteristics. Characteristics were no-normally distributed, used median and interquartile ranges (IQR) were used for description. Participants was divided into four groups based on the quartiles of the baseline TyG-BMI index, with the first quartile (Q1) being the lowest group and the fourth quartile (Q4) being the highest group. One-way ANOVA was used to test differences between groups for continuous variables. Kruskal-Wallis rank sum test was used to analyze differences between groups for continuous variables with non-normal distribution, and χ^2^ test was used for the analysis of categorical variables. In this study, the p-value for statistical tests is defined as “p ≤ 0.05 was considered to indicate a statistically significant difference”.

In this study, the follow-up time was calculated from the baseline to the last follow-up time, during which CVDs events or survival status were recorded. Univariate and multivariate analysis used Cox proportional hazard models to verify the relationship between the TyG-BMI index and CVDs, including their subtypes. The results were presented as hazard ratios (HR) and 95% confidence intervals (95%CI), and forest plots were used to visualize the results. Then, subgroup analyses were conducted by gender (male, female) and age (younger than 60 years, older than 60 years) using Cox proportional-hazards models to verify the relationship between the TyG-BMI index and CVDs within each subgroup, and the results were presented by HR and 95%CI.

We use a restricted cubic spline (with 4 knots) to explore the dose-response relationship between the TyG-BMI index and the incidence of CVDs. In this study, we introduced an unsupervised machine learning method, k-means clustering (22), to classify the TyG-BMI from 2011 to 2015. The rationale for using this method was its high computational efficiency, the ability to generate easily interpretable data points, and its strong visualization capabilities (23). The core idea of the K-means algorithm is to divide the dataset into K clusters, each represented by a centroid, in order to minimize the total squared distance between the data points within each cluster and the centroid, thereby partitioning the data into K clusters. To perform this operation, the following 4 steps are involved: a. Choose a predetermined number of clusters, K; b. Randomly select K data points as the initial centroids; c. Recalculate the centroid of each cluster by computing the average position of all data points within the cluster; d. Assign each data point to its nearest centroid. Steps c and d are repeated until the centroids no longer change or the preset maximum number of iterations is reached. All statistical analyses and plotting were performed by the R software (version 4.4.1, http://www.R-project.org/).

## Result

### Baseline characteristics and CVDs incidence of participants according to baseline TyG-BMI quartiles

A total of 7,072 subjects were included in the study (average age of 59.1 ± 9.3 years, 52.9% female), the demographic information and clinical characteristics of the study participants are presented in **Table 1**. At baseline, the average TyG index was 8.6 ± 0.6, triglyceride level was 103 mg/dL, FPG level was 108.4 ± 30.7 mg/dL, and the mean BMI was 23.4 ± 3.8 kg/m². Compared with other groups, the highest level group was more likely to be female, had a higher level of education, and lower proportions of current smoking and alcohol drinking. We observed that the levels of FBG, TG, TC and LDL-C were also higher in the fourth group, additionally, which had higher prevalence rates of hypertension, dyslipidemia, and diabetes. After a 9-year follow-up, there were 1,774 (25.1%) new cases of CVDs. The incidence rates of heart disease and stroke were 19.2% and 8.9%, respectively, with the highest incidence rates in the fourth group. Based on the quartiles of TyG-BMI index, the incidence of CHD and stroke indicated significant differences among the groups (P<0.001).

**Table 1:** Baseline characteristics of study population and incidence of CVDs in TyG-BMI quartiles.

Characteristic	Overall	Q1(102.4-174.9)	Q2(174.9-197.9)	Q3(197.9-225.6)	Q4(225.6-608.1)	p-value ^c^
**n**	7072	1768	1768	1768	1768	** **
Age(years) ^a^	59.1 ± 9.3	61.5 ± 9.9	59.2 ± 9.3	58.4 ± 8.9	57.2 ± 8.4	<0.001
**Gender**,n(%)						<0.001
Male	3,330 (47.1%)	1,022 (57.8%)	873 (49.4%)	774 (43.8%)	661 (37.4%)	
Female	3,742 (52.9%)	746 (42.2%)	895 (50.6%)	994 (56.2%)	1,107 (62.6%)	
**Marital status**,n(%)						<0.001
Married	6,271 (88.7%)	1,508 (85.3%)	1,555 (87.9%)	1,576 (89.1%)	1,632 (92.3%)	
Unmarried	801 (11.3%)	260 (14.7%)	213 (12.1%)	192 (10.9%)	136 (7.7%)	
**Education levels**,n(%)						<0.001
Primary school or below	4,952 (70.0%)	1,348 (76.2%)	1,270 (71.8%)	1,195 (67.6%)	1,139 (64.4%)	
Middle school	1,414 (20.0%)	292 (16.6%)	343 (19.4%)	362 (20.5%)	417 (23.5%)	
High school or above	706 (10.0%)	128 (7.2%)	155 (8.8%)	211 (11.9%)	212 (12.1%)	
**Smoking status**,n(%)						<0.001
Never smoker	4,321 (61.1%)	875 (49.5%)	1,044 (59.1%)	1,145 (64.8%)	1,257 (71.1%)	
Former smoker	578 (8.2%)	143 (8.1%)	122 (6.9%)	158 (8.9%)	155 (8.8%)	
Current smoker	2,173 (30.7%)	750 (42.4%)	602 (34.0%)	465 (26.3%)	356 (20.1%)	
**Drinking status**,n(%)						<0.001
Never drinking	4,111 (58.1%)	951 (53.8%)	991 (56.1%)	1,036 (58.6%)	1,133 (64.1%)	
Former drinker	546 (7.7%)	147 (8.3%)	130 (7.4%)	127 (7.2%)	142 (8.0%)	
Current drinker	2,415 (33.9%)	670 (37.9%)	647 (36.5%)	605 (34.2%)	493 (27.9%)	
FPG (mg/dl) ^a^	108.4 ± 30.7	99.6 ± 18.0	104.0 ± 23.6	108.6 ± 27.9	121.5 ± 42.9	<0.001
TC (mg/dl) ^a^	194.1 ± 37.8	184.7 ± 35.1	191.6 ± 37.5	196.9 ± 37.5	202.9 ± 38.7	<0.001
TG (mg/dl) ^b^	103 (73, 149)	73 (57, 93)	89 (69, 123)	112 (85, 156)	159 (117, 227)	<0.001
HDL-C(mg/dl) ^a^	51.8 ± 15.0	60.0 ± 15.4	54.8 ± 14.1	49.3 ± 13.2	42 .9 ± 11.4	<0.001
LDL-C(mg/dl) ^a^	117.7 ± 34.4	110.6 ± 31.1	117.9 ± 33.2	121.9 ± 33.8	120.6 ± 38.1	<0.001
UA (mg/dl) ^a^	4.4 ± 1.2	4.3 ± 1.2	4.3 ± 1.2	4.5 ± 1.3	4.7 ± 1.8	<0.001
SBP(mmHg) ^a^	130.0 ± 21.2	125.8 ± 21.6	127.3 ± 19.7	131.5 ± 21.4	135.3 ± 20.9	<0.001
DBP(mmHg) ^a^	75.6 ± 12.0	72.1 ± 11.5	74.0 ± 11.5	76 .6 ± 11.9	79.7 ± 11.9	<0.001
TyG index ^a^	8.6 ± 0.6	8.20 ± 0.4	8.5 ± 0.5	8.7 ± 0.5	9.2 ± 0.6	<0.001
BMI(kg/m²) ^a^	23.4 ± 3.8	19.4 ± 1.6	22.1 ± 1.3	24.2 ± 1.5	28.0 ± 3.5	<0.001
TyG-BMI ^a^	203.0 ± 39.9	158.5 ± 12.2	186.2 ± 6.6	210.9 ± 7.9	256.2 ± 30.8	<0.001
**Hypertension**,n(%)	2,701 (38.2%)	473 (26.8%)	547 (30.9%)	740 (41.9%)	941 (53.2%)	<0.001
**Dyslipidemia**,n(%)	2,865 (40.5%)	326 (18.4%)	541 (30.6%)	801 (45.3%)	1,197 (67.7%)	<0.001
**Diabetes**,n(%)	1,023 (14.5%)	99 (5.6%)	183 (10.4%)	258 (14.6%)	483 (27.3%)	<0.001
**CVDs**,n(%)	1,774 (25.1%)	337 (19.1%)	410 (23.2%)	453 (25.6%)	574 (32.5%)	<0.001
CHD,n(%)	1,355 (19.2%)	264 (14.9%)	311 (17.6%)	340 (19.2%)	440 (24.9%)	<0.001
Stroke,n(%)	627 (8.9%)	101 (5.7%)	141 (8.0%)	180 (10.2%)	205 (11.6%)	<0.001

^a^ Data are mean ± SD; ^b^ Data are median (IQR); ^c^ One-way ANOVA test; Kruskal-Wallis rank sum test; Pearson's Chi-squared test; Q1, First Quartile; Q2, Second Quartile; Q3, Third Quartile; Q4, Fourth Quartile; TyG index, triglyceride–glucose index; BMI, body mass index; TyG-BMI, triglyceride glucose-body mass index; FBG, fasting blood glucose; TC, total cholesterol; TG, triglyceride; HDL-C, high density lipoprotein cholesterol; LDL-C, low density lipoprotein cholesterol; SBP, systolic blood pressure; DBP, diastolic blood pressure; UA, uric acid; CVDs, cardiovascular diseases; CHD, coronary heart disease.

### Association of baseline TyG-BMI index with incident CVDs

In this study, during an average follow-up of 7.1 years, the incidence of CVDs was 25.1%, and the Q1 to Q4 groups were 19.1%, 23.2%, 25.6%, and 32.5%, respectively. CHD and stroke were 19.2% and 8.9%, and the incidence rates in Q1-Q4 were CHD (Q1,14.9%; Q2,17.6%; Q3,19.2%; Q4,24.9%) and stroke (Q1,5.7%; Q2,8.0%; Q3,10.2%; Q4,11.6%), respectively.

**Figure 2:**
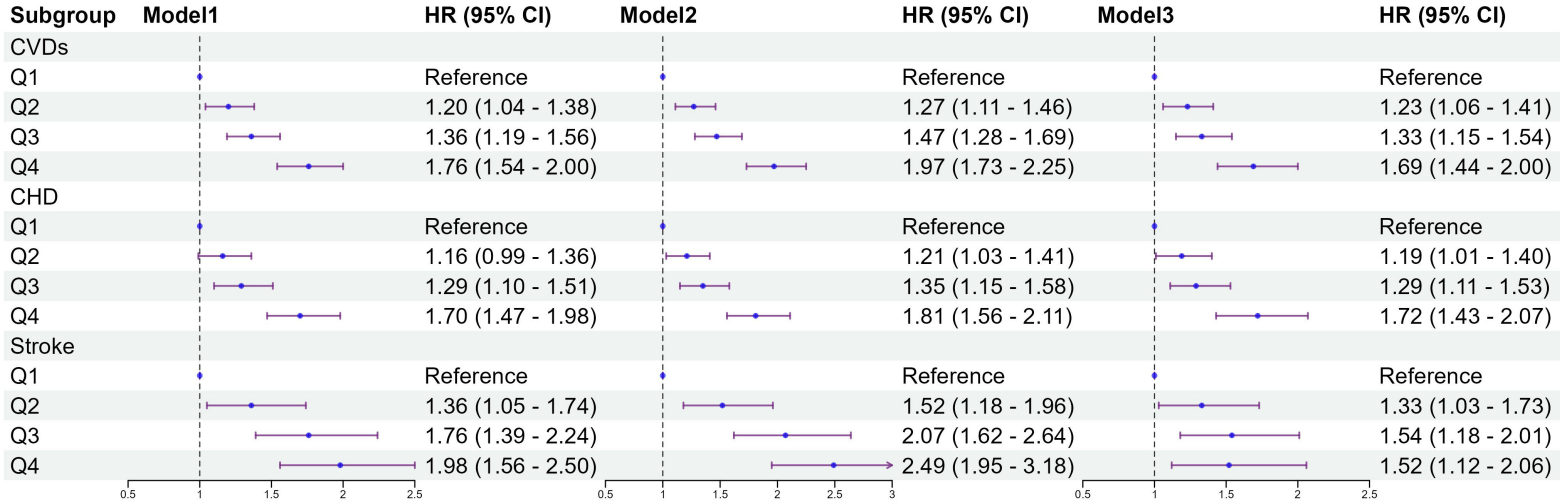
Association between quartiles of the baseline TyG-BMI with the incidence of CVDs. Model 1: unadjusted model; Model 2: adjusted for age, sex; Model 3: adjusted for age, sex, education, marital status, smoking status, alcohol consumption, FBG, TG, TC, LDL-C, HDL-C, hypertension, diabetes, dyslipidemia. HR, Hazard Ratio; 95% CI, 95% Confidence Interval; TyG index, triglyceride–glucose index; BMI, body mass index; TyG-BMI index, triglyceride–glucose index × body mass index; FBG, fasting blood glucose; TG, triglyceride; TC, total cholesterol; LDL-C, low-density lipoprotein cholesterol; HDL-C, high-density lipoprotein cholesterol; CVDs, cardiovascular diseases; CHD, coronary heart disease.

**Figure 2** shows the results of the Cox proportional hazards model. The results of the univariate Cox proportional hazards model analysis showed that compared to the lowest group (Q1), the highest TyG-BMI index levels group (Q4) was positively correlated with the risk of CVDs (HR 1.76; 95% CI 1.54–2.00). After covariates adjustment in model3, significant differences were observed in TyG-BMI index between Q2, Q3 and Q4 groups, compared with Q1 group. As the TyG-BMI index levels increased, compared with the lowest group, the adjusted hazard ratios (aHR) with 95% CI were 1.23(1.06-1.41), 1.33(1.15-1.54), and 1.69(1.44-2.00), respectively. Similarly, for the incidence of CHD and stroke events, the quartiles of the TyG-BMI index were consistent with the overall CVDs results; indicating that higher TyG-BMI index levels were associated with higher risks of CHD and stroke events, aHR, and 95% CI of Q4 vs Q1 in model 3 were 1.72(1.43-2.07), 1.52(1.12-2.06), respectively. Restricted cubic splines were used to explore the dose-response relationship between the TyG-BMI index and CVDs. The funnel plot presents the reference TyG-BMI index level as 198.36 with the non-linear P-value of 0.009. For subtypes, the reference value of the TyG-BMI index level were the same as for CVDs, with the nonlinear P < 0.001 for stroke (**Supplementary Figure S1**).

### Classes from the k-means clustering

**Figure 3:**
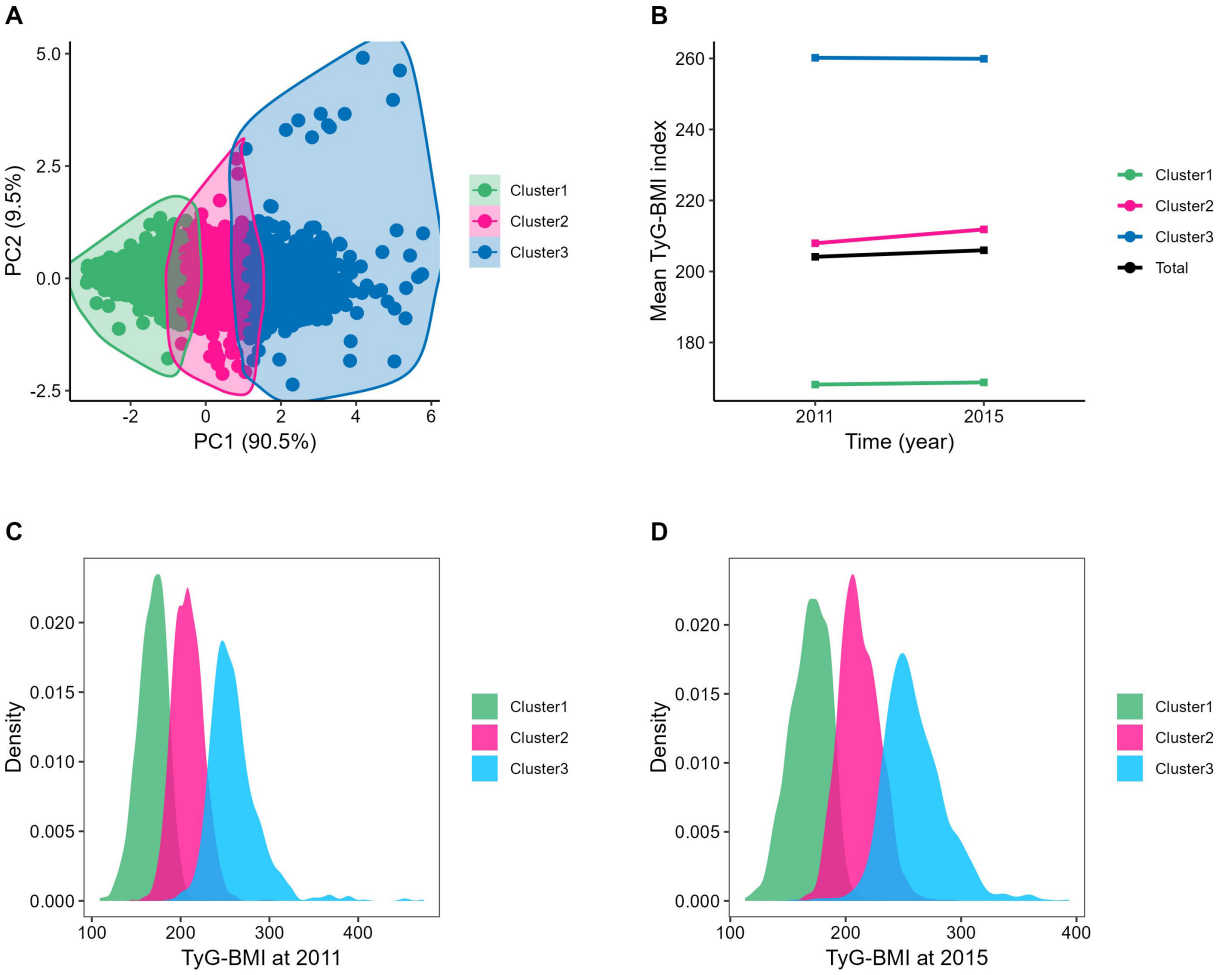
Clustering of the change in the TyG-BMI from 2011 to 2015. **(A)** Three clusters were found using the K-means method with Euclidean distance: the x- and y-axes are principal components of the change in the TyG-BMI; **(B)** The change of TyG-BMI from 2012 to 2015: Cluster1, the TyG-BMI changed from 168.16 ± 16.21 to168.77 ± 16.82; Cluster2, the TyG-BMI changed from 207.95 ± 16.92 to 211.84 ± 17.20; Cluster3, the TyG-BMI changed from 260.19 ± 29.60 to 259.90 ± 26.78; Total, the TyG-BMI changed from 204.14 ± 38.94 to 205.98 ± 38.59; **(C, D)** The distribution of TyG-BMI in 2011 or 2015: The normal distribution of TyG-BMI within each category was visible. PC, principal component, BMI, body mass index; TyG-BMI, triglyceride glucose-body mass index.

After 4 years of follow-up until the third wave of the survey, k-means clustering classified the TyG-BMI into three categories, as shown in **Figure 3A**. The total of TyG-BMI changed from 204.1 ± 38.9 in 2011 to 206.0 ± 38.6 in 2015 (n=4,151, P<0.001); and **Figure 3B** present the changes in each class: in class1 (n=1,510), the TyG-BMI changed from 168.2 ± 16.2 in 2011 to 168.8 ± 16.8 in 2015 (P=0.142), representing the participants with consistently low TyG-BMI levels; in class2 (n=1,794), the TyG-BMI changed from 208.0 ± 16.9 in 2011 to 211.8 ± 17.2 in 2015 (P<0.001), representing the participants with moderate TyG-BMI levels and a slowly increasing trend; in class3 (n=847), the TyG-BMI changed from 260.2 ± 29.6 in 2011 to 259.9 ± 26.8 in 2015 (P<0.001), representing the participants with the highest TyG-BMI levels and a slowly decreasing trend. The distribution of TyG-BMI in 2011 or 2015is presented in **Figures 3C, D**.

### Baseline characteristics of participants according to change of TyG-BMI

Excluding samples without blood information, there were a total of 4,151 participants (average age of 58.7 ± 8.7 years, 54.3% female) in 2015. After K-means clustering analysis, the comparison results by different categories are shown in **Supplementary Table S1**. Compared to the former crowd, this population exhibited higher levels of FBG (100.7 ± 30.4mg/dL), TG [112(81,163)mg/dL], BMI (23.7 ± 3.6kg/m²), and TyG-BMI (206.0 ± 38.6), along with a higher incidence of CVDs (1137,27.4%). The differences in covariates such as gender, marry status, and drinking status across the category groups were statistically significant (P<0.001). Based on the different categories, the incidence of CVD was 27.4% in total, with classes1 to 3 were 21.7%, 28.4%, 35.4%, respectively. For CHD and stroke, the incidences were 21.5% and 9.3%, respectively, with classes 1 to 3 showed incidences of CHD (16.9%, 22.1%, 28.3%) and stroke (6.8%, 9.9%, 12.6%). There were differences in the incidence of CVDs, coronary heart disease and stroke between groups based on TyG-BMI categories (P<0.001).

### Hazard ratios for incident CVDs according to change of TyG-BMI

**Figure 4:**
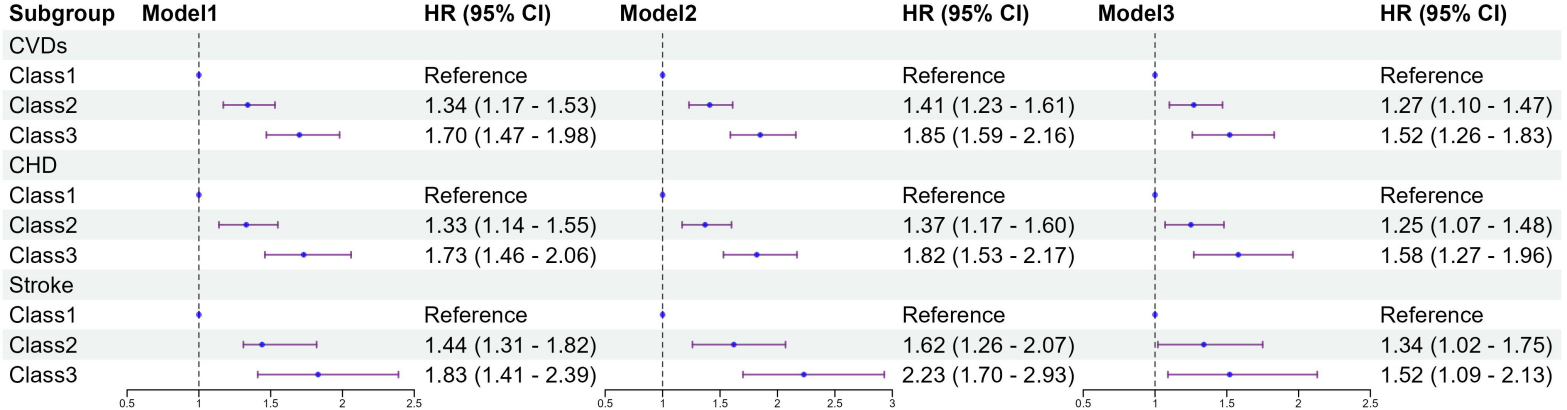
Association between classes of the baseline TyG-BMI with the incidence of CVDs. Model 1: unadjusted model; Model 2: adjusted for age, sex; Model 3: adjusted for age, sex, education, marital status, smoking status, alcohol consumption, FBG, TG, TC, LDL-C, HDL-C, hypertension, diabetes, dyslipidemia. HR, Hazard Ratio; 95% CI, 95% Confidence Interval; TyG index, triglyceride–glucose index; BMI, body mass index; TyG-BMI index, triglyceride–glucose index × body mass index; FBG, fasting blood glucose; TG, triglyceride; TC, total cholesterol; LDL-C, low-density lipoprotein cholesterol; HDL-C, high-density lipoprotein cholesterol; CVDs, cardiovascular diseases; CHD, coronary heart disease.

**Figure 4** shows the results of the Cox proportional hazards model. Compared to class 1, class3 had a higher risk of CVDs (HR 1.70; 95% CI 1.47–1.98). After multivariable adjustment in model 3, compared to class1, the adjusted hazard ratios (aHR) with 95% CI for class2 and class3 were 1.27(1.10-1.47), and 1.52(1.26-1.83). Both CHD and stroke show similar results across different categories, aHR, and 95% CI of class3 vs class1 were 1.58(1.27-1.96), 1.52(1.09-2.13), respectively. Restricted cubic splines were used to explore the dose-response relationship between the TyG-BMI and CVDs. The funnel plot presents the reference TyG-BMI index level as 199.26 and the non-linear P-value of 0.005. For subtypes, the reference value of the TyG-BMI index level were the same as for CVDs, with the nonlinear P-value of 0.007 for CHD (**Supplementary Figure S2**).

### ROC curve analysis of TyG-BMI, TyG, BMI prediction CVDs

**Figure 5:**
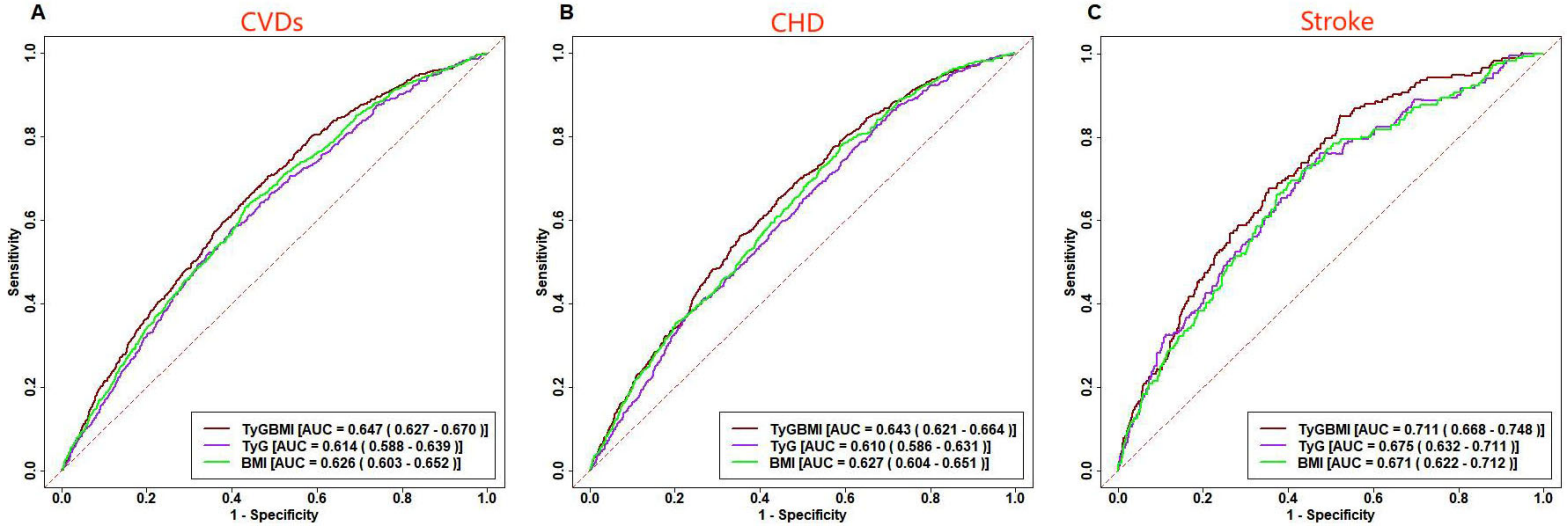
ROC curve analysis of TyG-BMI, TyG, BMI prediction CVDs. Receiver operating characteristic curves for baseline TyG-BMI, TyG, BMI predicting CVDs **(A)**, CHD **(B)** and Stroke **(C)**.

ROC curve analysis indicates that TyG-BMI had the highest predictive ability for the incidence of CVDs among the three indicators, with an AUC of 0.647, slightly overmatching to TyG (AUC = 0.614) and BMI (AUC = 0.626) in the multivariate model (**Figure 5**).

In the multivariate model, the superior predictive performance of TyG-BMI compared to TyG and BMI was also observed in CVDs subtypes, with an AUC of 0.643 for predicting CHD and an AUC of 0.711 for predicting stroke.

### Subgroup analysis

The association of the variability of TyG-BMI and TyG-BMI with CVDs incidence stratified by different risk factors that was presented by **Tables 2** and **3**. After adjusting for confounding potential factors, smoking and marital status moderated the relationship between TyG-BMI change categories and cardiovascular events; and age, smoking and marital status moderated the association of TyG-BMI and cardiovascular events.

**Table 2:** Association of different categories of change in TyG-BMI with the risk of CVDs stratified by different factors.

Subgroup	Change in the TyG-BMI, HR (95%CI)			
	Class1 (1510)	Class2 (1794)	Class3 (847)	p for interaction
Age				0.34
<60	Reference	1.26 (1.01,1.57)	1.52 (1.15,2.00)	
≥60	Reference	1.26 (1.04,1.52)	1.40 (1.08,1.83)	
Gender				0.6
Male	Reference	1.25 (1.01,1.56)	1.74 (1.28,2.37)	
Female	Reference	1.25 (1.03,1.52)	1.36 (1.07,1.72)	
Marital status				0.007
Married	Reference	1.25 (1.07,1.46)	1.55 (1.27,1.89)	
Unmarried	Reference	1.32 (0.88,1.98)	0.82 (0.42,1.61)	
Education levels				0.901
Primary school or below	Reference	1.23 (1.04,1.44)	1.41 (1.14,1.75)	
Middle school	Reference	1.35 (0.92,1.99)	1.52 (0.93,2.50)	
High school or above	Reference	1.24 (0.75,2.06)	1.82 (0.91,3.61)	
Smoking status				0.033
Never smoker	Reference	1.21 (1.01,1.46)	1.48 (1.17,1.86)	
Former smoker	Reference	0.74 (0.44,1.22)	1.19 (0.64,2.20)	
Current smoker	Reference	1.59 (1.23,2.06)	1.50 (0.99,2.28)	
Drinking status				0.984
Never drinking	Reference	1.23 (1.02,1.49)	1.40 (1.10,1.77)	
Former drinker	Reference	1.19 (0.73,1.92)	1.58 (0.84,2.97)	
Current drinker	Reference	1.33 (1.03,1.72)	1.64 (1.14,2.35)	
Hypertension				0.77
Yes	Reference	1.33 (1.06,1.67)	1.52 (1.16,1.99)	
No	Reference	1.18 (0.98,1.43)	1.37 (1.04,1.82)	
Dyslipidemia				0.826
Yes	Reference	1.35 (1.05,1.74)	1.60 (1.20,2.14)	
No	Reference	1.17 (0.98,1.41)	1.37 (1.04,1.81)	
Diabetes				0.979
Yes	Reference	1.26 (0.81,1.98)	1.31 (0.81,2.12)	
No	Reference	1.26 (1.08,1.47)	1.52 (1.23,1.88)	

HR, Hazard Ratio; 95% CI, 95% Confidence Interval; TyG-BMI index, triglyceride–glucose index × body mass index; CVDs, cardiovascular diseases; All models adjusted for age, sex, education, marital status, smoking status, alcohol consumption, FBG, TG, TC, LDL-C, HDL-C, hypertension, diabetes, dyslipidemia.

**Table 3:** Association of TyG-BMI with the risk of CVDs stratified by different factors.

Subgroup	TyG-BMI, HR (95%CI)				
	Q1 (102.4-174.9)	Q2 (174.9,197.9)	Q3 (197.9,225.6)	Q4 (225.6,608.1)	p for interaction
Age					0.043
<60	Reference	1.22 (0.97,1.53)	1.49 (1.19,1.88)	1.64 (1.28,2.11)	
≥60	Reference	1.23 (1.03,1.48)	1.14 (0.94,1.39)	1.59 (1.27,1.98)	
Gender					0.965
Male	Reference	1.25 (1.02,1.53)	1.33 (1.07,1.66)	1.68 (1.31,2.16)	
Female	Reference	1.18 (0.97,1.45)	1.27 (1.04,1.55)	1.57 (1.26,1.95)	
Marital status					0.042
Married	Reference	1.29 (1.10,1.51)	1.34 (1.14,1.57)	1.70 (1.42,2.02)	
Unmarried	Reference	0.90 (0.63,1.30)	1.08 (0.73,1.59)	1.10 (0.65,1.88)	
Education levels					0.852
Primary school or below	Reference	1.17 (0.99,1.37)	1.23 (1.04,1.46)	1.52 (1.26,1.84)	
Middle school	Reference	1.25 (0.87,1.80)	1.28 (0.88,1.87)	1.78 (1.19,2.66)	
High school or above	Reference	1.77 (1.04,3.01)	1.91 (1.12,3.27)	2.17 (1.17,4.00)	
Smoking status					0.025
Never smoker	Reference	1.24 (1.02,1.50)	1.36 (1.12,1.65)	1.73 (1.40,2.14)	
Former smoker	Reference	0.87 (0.57,1.34)	0.72 (0.46,1.11)	1.22 (0.76,1.97)	
Current smoker	Reference	1.36 (1.07,1.74)	1.49 (1.14,1.94)	1.53 (1.10,2.14)	
Drinking status					0.091
Never drinking	Reference	1.23 (1.01,1.49)	1.35 (1.11,1.65)	1.68 (1.36,2.07)	
Former drinker	Reference	0.77 (0.50,1.19)	0.65 (0.40,1.07)	0.92 (0.55,1.55)	
Current drinker	Reference	1.38 (1.08,1.76)	1.45 (1.12,1.88)	1.72 (1.26,2.33)	
Hypertension					0.617
Yes	Reference	1.33 (1.06,1.68)	1.31 (1.04,1.65)	1.64 (1.28,2.09)	
No	Reference	1.14 (0.96,1.37)	1.29 (1.06,1.57)	1.53 (1.21,1.94)	
Dyslipidemia					0.71
Yes	Reference	1.37 (1.03,1.81)	1.41 (1.07,1.85)	1.73 (1.31,2.29)	
No	Reference	1.13 (0.96,1.34)	1.19 (0.99,1.44)	1.51 (1.21,1.89)	
Diabetes					0.085
Yes	Reference	2.20 (1.29,3.73)	1.65 (0.97,2.81)	2.17 (1.28,3.67)	
No	Reference	1.17 (1.01,1.36)	1.31 (1.12,1.54)	1.61 (1.34,1.93)	

HR, Hazard Ratio; 95% CI, 95% Confidence Interval; TyG-BMI index, triglyceride–glucose index × body mass index; CVDs, cardiovascular diseases; All models adjusted for age, sex, education, marital status, smoking status, alcohol consumption, FBG, TG, TC, LDL-C, HDL-C, hypertension, diabetes, dyslipidemia.

## Discussion

Among 7,072 middle-aged and elderly participants followed from 2011 to 2020, a significant relationship was observed between different quartiles of baseline TyG-BMI index and its variation over time with the incidence of CVDs in the cohort study. Furthermore, the association between the TyG-BMI index and CVDs remained significant even after adjusting for multiple CVDs risk factors. Additionally, the TyG-BMI index showed a similar positive correlation in assessing the risk of CVDs, CHD, and stroke. These findings highlight the predictive value of TyG-BMI levels and their patterns of change for predicting cardiovascular and cerebrovascular events in middle-aged and elderly individuals.

Based on our research findings, a higher TyG-BMI index compared to lower levels is associated with an increased overall risk of cardiovascular diseases in middle-aged and elderly individuals by approximately 20% to 60%. The impact of TyG-BMI index is even greater for CHD, with the risk attain 1.7 times, while the risk for stroke also increased ranging from 30% to 50%. Our research findings indicate that the baseline TyG-BMI index can be used to identify individuals at risk for various cardiovascular diseases. If the TyG-BMI exceeds 198.36, individuals should be advised to take this as a warning and pay close attention to their cardiovascular health. Previous studies have indicated a positive correlation between TyG-BMI and CVDs (21, 24, 25), which is consistent with our findings. In a 10-year prospective cohort study involving 11,016 participants, it was found that TyG-BMI can reflect insulin resistance, and higher levels of TyG-BMI increased the risk of CVDs by 1.38 times (24). We confirmed this finding using a national cohort and analyzed different types of cardiovascular diseases in our study. Another recent study indicated that higher levels of TyG-BMI may be associated with an increased incidence of congestive heart failure, myocardial infarction, angina pectoris, and coronary artery disease (25). These studies are almost always consistent with our findings, and all demonstrate a significant correlation between TyG-BMI and CVDs events. Other related researches, restricted cubic spline curves indicated that a TyG-BMI index between 190 and 200 could serve as a reference for assessing disease risk (26, 27). These findings are generally consistent with our results.

We found that the TyG-BMI persistence group was at higher risk of CVDs, CHD, and stroke, compared to middle-aged and older adults in the group with lower levels of TyG-BMI index in our study. In previous studies, most focused on the relationship between the variation of the single TyG index and CVDs. A 10-year prospective cohort study indicated that the trajectory of TyG changes in the moderate-stable group was associated with the progression of carotid artery stenosis (CAS), while no association was observed in the high-increasing group (28). The results are inconsistent with ours. Results from the Kailuan study suggest that cumulative TyG is associated with an increased risk of CVDs (29). Another study indicated that variations in the TyG-WHtR are associated with the occurrence of CVDs, and that the progression of CVDS was significantly associated with moderate and high levels (30). These studies, as well as ours, provide evidence for the long-term monitoring of TyG-BMI variation and CVDs progression.

We performed subgroup analyses to validate whether our study applies to different populations. The results of the subgroup analyses were generally consistent with the main findings, except for smokers and unmarried individuals. A previous cohort study showed that living alone was associated with a higher risk of developing CVDs (31). Additionally, a meta-analysis indicated that being unmarried was linked to an increased risk of CVDs (32). Moreover, multiple studies have shown that smoking increases the risk of developing CVDs (33–35). These findings suggest that smoking and being unmarried are associated with a higher risk of CVDs, indicating that TyG-BMI may not effectively predict high-risk CVD individuals within these populations.

IR is a systemic condition that affects multiple organs and insulin-regulatory pathways. It is characterized by a diminished insulin response despite elevated insulin levels (36). Previous studies have shown that IR is an important risk factor for CVDs (37, 38). IR impairs glycogen synthesis and protein catabolism in skeletal muscle and inhibits lipoprotein lipase activity in adipocytes, leading to increased release of free fatty acids(FFA) and inflammatory cytokines. The elevated FFA could accumulate as lipids, causing their influx into the liver and other organs, further exacerbating IR and contributing to systemic IR (39, 40). At the same time, overweight and obesity, as key factors in metabolic disorders, not only increase the risk of CVDs but also lead to IR by mediating the flow of FFA due to the accumulation of body fat (41, 42). HEC is considered the gold standard for assessing insulin resistance; however, its complex and costly procedure limits its widespread clinical use (14–16). There is an urgent need for a simpler alternative method. Recently, TyG-BMI index has emerged as a novel alternative indicator for IR. Due to its inclusion of FBG, TG, and BMI, it offers greater applicability (43–46).

There are several advantages to this study. Firstly, we utilized data from a nationwide prospective cohort study of middle-aged and elderly individuals. Secondly, compared to the gold standard for IR, TyG-BMI provides an economical, simple, and effective alternative method. Additionally, we not only conducted analyses in middle-aged and elderly populations, but also performed subgroup analyses across different demographic groups. Finally, we used repeated measures data to explore the relationship between changes in TyG-BMI and the incidence of CVDs and its subtypes. We also adjusted for multiple potential confounding factors to minimize their impact on the results.

However, our research also has some limitations. Firstly, our study is observational and lacks intervention and randomization, and could not completely exclude all unmeasurable confounders. Due to the nature of observational studies, we may not be able to establish causal relationships, which need to be verified in future research. Secondly, our study population was limited to individuals over 45 years old, and cannot be better generalized to other age groups. Additionally, CVDs were primarily defined as self-reported CHD and stroke in the CHARLS data, which may be making it difficult to verify their accuracy. However, previous studies have shown that self-reports are generally consistent with medical diagnosis records, indicating that the risk of misreporting bias is minimal (47).

## Conclusion

In the prospective study of middle-aged and elderly populations, we demonstrated that high levels of TyG-BMI is an independent risk factor for CVDs, CHD, and stroke, whether due to elevated baseline TyG-BMI levels or fluctuations that remain at high levels. Additionally, TyG-BMI could serve as a simple, accessible, cost-effective, and relatively reliable alternative indicator for IR. Therefore, detecting long-term changes in TyG-BMI could aid in the early identification of high-risk individuals, thereby preventing the occurrence of various CVDs.

## Data Availability

Publicly available datasets were analyzed in this study. This data can be found here: http://charls.pku.edu.cn.

## Supplementary material

Supplementary Figure 1 Restricted cubic spline plots for associations of baseline TyG-BMI index levels with CVDs and subtypes among 7,072 participants. HRs and 95%CIs for A(CVDs), B(CHD), and C(Stroke) based on restricted cubic splines for baseline TyG index; HRs and 95%CIs were calculated using Cox proportional-hazards models after adjustment for age. HRs, Hazard Ratios; 95%CIs, 95% Confidence Intervals; CVDs, cardiovascular diseases; CHD, coronary heart disease.

Supplementary Figure 2 Restricted cubic spline plots for associations of baseline TyG-BMI index levels with CVDs and subtypes among 4,151 participants. HRs and 95%CIs for A(CVDs), B(CHD), and C(Stroke) based on restricted cubic splines for baseline TyG index; HRs and 95%CIs were calculated using Cox proportional-hazards models after adjustment for age. HRs, Hazard Ratios; 95%CIs, 95% Confidence Intervals; CVDs, cardiovascular diseases; CHD, coronary heart disease.
