# Supplementary figures and images for: Relationship between triglyceride glucose-body mass index baselines and variation with future cardiovascular diseases risk in the middle-aged and elderly individuals
Source: Front Endocrinol (Lausanne). 2025 Jan 27;16:1514660. doi: 10.3389/fendo.2025.1514660 (PMC11807823; doi:10.3389/fendo.2025.1514660)

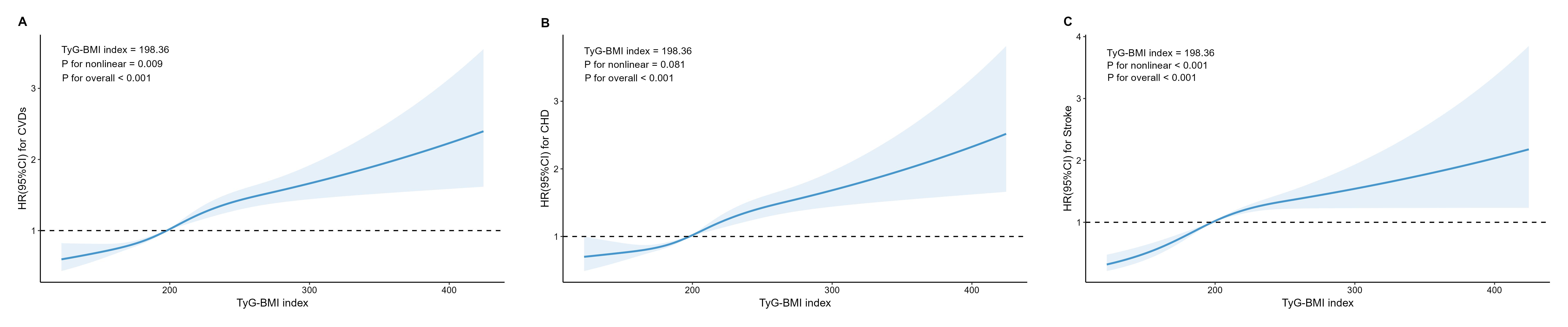

Supplement: Supplementary Figure 1 — Restricted cubic spline plots for associations of baseline TyG-BMI index levels with CVDs and subtypes among 7,072 participants. HRs and 95%CIs for A(CVDs), B(CHD), and C(Stroke) based on restricted cubic splines for baseline TyG index; HRs and 95%CIs were calculated using Cox proportional-hazards models after adjustment for age. HRs, Hazard Ratios; 95%CIs, 95% Confidence Intervals; CVDs, cardiovascular diseases; CHD, coronary heart disease. [file Image1.jpeg]

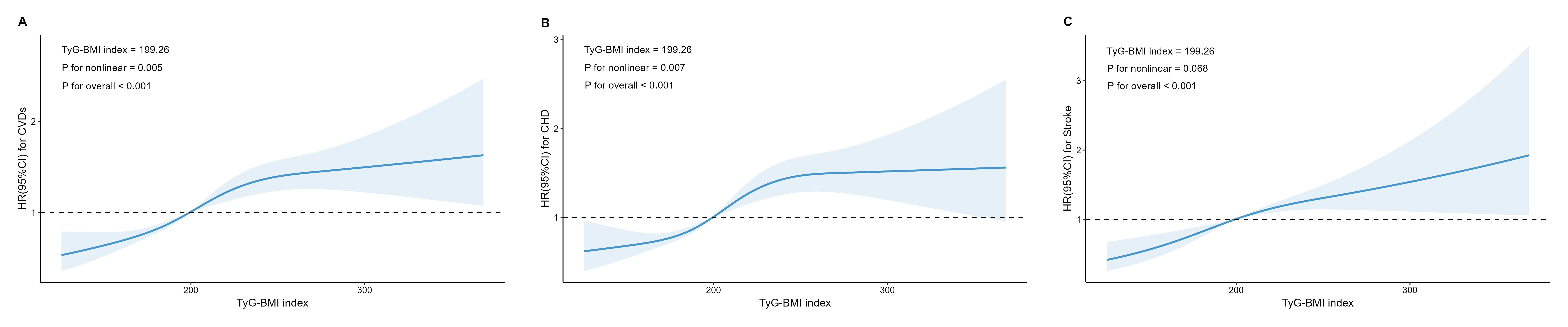

Supplement: Supplementary Figure 2 — Restricted cubic spline plots for associations of baseline TyG-BMI index levels with CVDs and subtypes among 4,151 participants. HRs and 95%CIs for A(CVDs), B(CHD), and C(Stroke) based on restricted cubic splines for baseline TyG index; HRs and 95%CIs were calculated using Cox proportional-hazards models after adjustment for age. HRs, Hazard Ratios; 95%CIs, 95% Confidence Intervals; CVDs, cardiovascular diseases; CHD, coronary heart disease. [file Image2.jpeg]
