# Supplementary material for: Relationship between triglyceride glucose-body mass index baselines and variation with future cardiovascular diseases risk in the middle-aged and elderly individuals
Source: Front Endocrinol (Lausanne). 2025 Jan 27;16:1514660. doi: 10.3389/fendo.2025.1514660 (PMC11807823; doi:10.3389/fendo.2025.1514660)
Supplement: Supplementary file 3 [file Table1.docx]

Table S1. Baseline characteristics of participants by K-means clustering analysis

| **Characteristic** | **Overall** | **Class1** | **Class2** | **Class3** | **p-value** ^c^ |
| --- | --- | --- | --- | --- | --- |
| **n** | **4151** | **1510** | **1794** | **847** |  |
| Age(years) ^a^ | 58.7 ± 8.7 | 60.6 ± 9.0 | 58.1 ± 8.4 | 56.6 ± 7.9 | <0.001 |
| **Gender**,n(%) |  |  |  |  | <0.001 |
| Male | 1,898 (45.7%) | 856 (56.7%) | 758 (42.3%) | 284 (33.5%) |  |
| Female | 2,253 (54.3%) | 654 (43.3%) | 1,036 (57.7%) | 563 (66.5%) |  |
| **Marry status**,n(%) |  |  |  |  | <0.001 |
| Married | 3,715 (89.5%) | 1,319 (87.4%) | 1,605 (89.5%) | 791 (93.4%) |  |
| Unmarried | 436 (10.5%) | 191 (12.6%) | 189 (10.5%) | 56 (6.6%) |  |
| **Education levels**,n(%) |  |  |  |  | <0.001 |
| Primary school or below | 2,935 (70.7%) | 1,133 (75.1%) | 1,225 (68.3%) | 577 (68.1%) |  |
| Middle school | 840 (20.2%) | 272 (18.1%) | 381 (21.2%) | 187 (22.1%) |  |
| High school or above | 376 (9.1%) | 105 (6.8%) | 188 (10.5%) | 83 (9.8%) |  |
| **Smoking status**,n(%) |  |  |  |  | <0.001 |
| Never smoker | 2,587 (62.3%) | 779 (51.6%) | 1,184 (66.0%) | 624 (73.6%) |  |
| Former smoker | 320 (7.7%) | 106 (7.0%) | 150 (8.4%) | 64 (7.6%) |  |
| Current smoker | 1,244 (30.0%) | 625 (41.4%) | 460 (25.6%) | 159 (18.8%) |  |
| **Drinking status**,n(%) |  |  |  |  | <0.001 |
| Never drinking | 2,427 (58.5%) | 795 (52.6%) | 1,072 (59.8%) | 560 (66.1%) |  |
| Former drinker | 314 (7.6%) | 121 (8.1%) | 130 (7.2%) | 63 (7.4%) |  |
| Regular drinker | 1,410 (33.9%) | 594 (39.3%) | 592 (33.0%) | 224 (26.5%) |  |
| TC (mg/dl) ^a^ | 194.0 ± 37.8 | 186.3 ± 36.1 | 195.7 ± 37.2 | 204.1 ± 39.1 | <0.001 |
| HDL-C(mg/dl) ^a^ | 51.5 ± 14.9 | 58.4 ± 15.9 | 49.5 ± 12.7 | 43.3 ± 12.1 | <0.001 |
| LDL-C(mg/dl) ^a^ | 117.7 ± 34.3 | 111.8 ± 31.4 | 121.0 ± 34.1 | 121.2 ± 37.9 | <0.001 |
| UA (mg/dl) ^a^ | 4.4 ± 1.2 | 4.2 ± 1.2 | 4.4 ± 1.2 | 4.6 ± 1.3 | <0.001 |
| SBP (mmHg) ^a^ | 129.4 ± 20.8 | 125.3 ± 19.9 | 130.0 ± 20.7 | 135.3 ± 21.1 | <0.001 |
| DBP (mmHg) ^a^ | 75.5 ± 11.9 | 72.27 ± 11.2 | 76.01 ± 11.7 | 80.19 ± 12.1 | <0.001 |
| FPG_2011_ (mg/dl) ^a^ | 108.6 ± 30.59 | 102.4 ± 21.9 | 108.0 ± 28.4 | 120.8 ± 42.6 | <0.001 |
| TG_2011_ (mg/dl) ^b^ | 103 (73, 149) | 77 (60, 105) | 107 (81, 150) | 159 (115, 231) | <0.001 |
| TyG_2011_^a^ | 8.7 ± 0.6 | 8.3 ± 0.5 | 8.7 ±0.5 | 9.2 ± 0.6 | <0.001 |
| BMI_2011_ (kg/m²) ^a^ | 23.5 ± 3.7 | 20.3 ± 1.8 | 24.0 ± 1.8 | 28.5 ± 3.3 | <0.001 |
| TyG-BMI_2011_^a^ | 204.1 ± 38.9 | 168.2 ± 16.2 | 208.0 ± 16.9 | 260.2 ± 29.6 | <0.001 |
| FPG_2015_ (mg/dl) ^a^ | 100.7 ± 30.4 | 93.3 ± 19.7 | 100.1 ± 25.6 | 115.4 ± 46.1 | <0.001 |
| TG_2015_ (mg/dl) ^b^ | 112 (81, 163) | 83 (67, 109) | 120 (91, 165) | 174 (129, 247) | <0.001 |
| TyG_2015_^a^ | 8.7 ± 0.6 | 8.3 ± 0.4 | 8.7 ± 0.5 | 9.2 ± 0.6 | <0.001 |
| BMI_2015_ (kg/m²) ^a^ | 23.7 ± 3.6 | 20.4 ± 1.9 | 24.3 ± 1.9 | 28.3 ± 2.8 | <0.001 |
| TyG-BMI_2015_^a^ | 206.0± 38.6 | 168.8 ± 16.8 | 211.8 ± 17.2 | 259.9 ± 26.8 | <0.001 |
| **Hypertension**,n(%) | 1,573 (37.9%) | 394 (26.1%) | 713 (39.7%) | 466 (55.1%) | <0.001 |
| **Dyslipidemia**,n(%) | 1,732 (41.7%) | 346 (22.9%) | 797 (44.4%) | 589 (69.5%) | <0.001 |
| **Diabetes**,n(%) | 603 (14.5%) | 122 (8.1%) | 250 (13.9%) | 231 (27.3%) | <0.001 |
| **CVDs**,n(%) | 1,137 (27.4%) | 327 (21.7%) | 510 (28.4%) | 300 (35.4%) | <0.001 |
| CHD,n(%) | 892 (21.5%) | 255 (16.9%) | 397 (22.1%) | 240 (28.3%) | <0.001 |
| Stroke,n(%) | 387 (9.3%) | 103 (6.8%) | 177 (9.9%) | 107 (12.6%) | <0.001 |

^a^ Data are mean ± SD; ^b^ Data are median (IQR); ^c^ One-way ANOVA test; Kruskal-Wallis rank sum test; Pearson's Chi-squared test; TyG index, triglyceride–glucose index; BMI, body mass index; TyG-BMI, triglyceride glucose-body mass index; FBG, fasting blood glucose; TC, total cholesterol; TG, triglyceride; HDL-C, high density lipoprotein cholesterol; LDL-C, low density lipoprotein cholesterol; SBP, systolic blood pressure; DBP, diastolic blood pressure; UA, uric acid; CVDs, cardiovascular diseases; CHD, coronary heart disease.
